# Vaccines for the Leishmaniases: Proposals for a Research Agenda

**DOI:** 10.1371/journal.pntd.0000943

**Published:** 2011-03-29

**Authors:** The Working Group on Research Priorities for Development of Leishmaniasis Vaccines, Carlos Henrique Nery Costa, Nathan C. Peters, Sandra Regina Maruyama, Eldo Cardoso de Brito, Isabel Kinney Ferreira de Miranda Santos

**Affiliations:** 1 Natan Portella Institute for Tropical Diseases, Federal University of Piauí, Teresina, Brazil; 2 Laboratory of Parasitic Diseases, National Institute of Allergy and Infectious Disease, National Institutes of Health, Bethesda, Maryland, United States of America; 3 Laboratory of Immunoparasitology, Department of Biochemistry and Immunology, Ribeirão Preto School of Medicine, University of São Paulo, Ribeirão Preto, Brazil; 4 National Center for Genetic Resources and Biotechnology, Brazilian Enterprise for Agricultural Research, Brasília, Brazil; Institut Pasteur de Tunis, Tunisia

The International Symposium on Leishmaniasis Vaccines, held in Olinda, Brazil, on March 9–11, 2009, congregated international experts who conduct research on vaccines against the leishmaniases. The questions that were raised during that meeting and the ensuing discussions are compiled in this report and may assist in guiding a research agenda. A group to further discussion on issues raised in this policy platform has been set up at http://groups.google.com/group/leishvaccines-l.

## The Impact of the Leishmaniases and Perspectives for Anti-*Leishmania* Vaccines

The leishmaniases are responsible for the second-highest number of deaths due to parasitic infection globally and are overwhelmingly associated with poverty [1]. They have an estimated prevalence of 12 million humans infected and cause a burden estimated at 2,357,000 disability-adjusted life years [1]. Visceral leishmaniasis (VL) is almost always fatal if not treated, and morbidity caused by the cutaneous leishmaniases (CLs) is also important. Treatments for all forms of leishmaniases are few, toxic, and/or expensive, and, furthermore, drug resistance is on the rise [2]. Canids are significant zoonotic reservoirs for human VL (their role as a reservoir for other species of *Leishmania* is not fully defined [3]) and a cause of concern for owners of dogs; they are also important sources of knowledge about the human counterparts of this infection. Leishmaniases are vector-borne diseases, and the impact of global warming on the geographical distribution of parasite-infected sand flies suggests that the leishmaniases may become a widespread and significant problem for public health. Political and socioeconomic changes may have an even more important role than global warming on the changing epidemiology of the leishmaniases, as has been argued for tick-borne diseases in Europe [4]. Indeed, in the last 20 years in Brazil, the epidemiological pattern of VL has changed from a sporadic disease of rural areas to one occurring in peri-urban epidemics that affect all socioeconomic strata, and with a trend towards increasing mortality [5]. The European Centre for Disease Prevention and Control lists the leishmaniases among the ten vector-borne diseases that have the greatest potential to affect European inhabitants [6]. The leishmaniases are, so far, not preventable diseases, and their epidemiological profile is shifting. This current situation demands novel instruments for treatment and control.

The feasibility of controlling the leishmaniases with a vaccine, a likely cost-effective approach for their control in many epidemiological situations, is attested to by the fact that most individuals who were once infected and recovered are resistant to overt clinical manifestations upon re-infections [7]. There are, however, still many challenges to overcome before effective vaccines for prevention of the leishmaniases become a reality. The centuries-old practice of leishmanization, which uses inoculation of live, virulent *Leishmania major* parasites at cosmetically acceptable sites of the body, affords lifelong protection against CL and represents the only efficacious vaccine for any disease of this complex [8]; its correlates of protection should be exhaustively explored.

Regarding product profiles, if a live vaccine proves to be the only successful route to controlling the leishmaniases, issues of logistics will certainly arise. However, this aspect has not prevented two other live vaccines to eradicate one disease (smallpox) and almost eradicate another (polio). Product profiles, in turn, directly affect research strategies and clinical development, but should not abort research on useful antigens that otherwise could compose a “second best” product profile. At present there is no consensus about the product profile for vaccines against the leishmaniases, except the minimum requirements for neglected parasitic diseases: safe, effective, and inexpensive.

## Mechanisms of Protective Immunity

In animal models, immunity against *Leishmania* has historically been cast within the Th1/Th2 paradigm. Cellular immunity is considered to be the key mediator of resistance by means of IFN-γ, which upregulates the production of nitric oxide, leading to oxidative burst in phagocytes that harbor these intracellular pathogens. However, evidence of Th2 and T regulatory responses in resistant mice, and progressive disease in resistant strains that have defects in Th1 differentiation but do not default to a Th2 pathway, has led to a modification of this paradigm to state that a Th2 response does not promote disease, but the absence of Th1 immunity does [9]. In addition, the discovery of IL-10-mediated regulation of protective IFN-γ-producing cells has shown that a highly regulated Th1 response can also lead to susceptibility [10]. The Th1 immune response can also be associated with immunopathology; for example, development of lesions in human tegumentary leishmaniasis is due to activation of type 1 immune responses. Moreover, individuals infected with *L. braziliensis* but who do not develop disease produce less type 1 cytokines than patients with cutaneous lesions [11]. Studies must be done to determine whether such individuals did not develop disease because they had small Th1 immune responses or because they received a low dose of antigen.

The precise role of antibodies in conferring resistance to infections with *Leishmania* is controversial and needs to be reappraised. The role of antibodies in tuberculosis, caused by an intracellular pathogen, was also controversial. However, recent work shows that antibodies participate in important effector mechanisms in the response against tuberculosis [12]. Antibodies also regulate the outcome of immune responses through different types of Fc receptors [13]. Animals lacking B cells are resistant to infections with *Leishmania* [14], and the elevated levels of IgG seen in human and canine VL suggest that antibodies are not relevant for protection in the absence of an appropriate cellular response. More recent work, however, supports a role of antibodies in protection against *Leishmania*. Natural antibodies in normal human sera have been shown to very rapidly kill infective culture promastigotes by the classical pathway of complement [15]. Sera from dogs vaccinated against *L. infantum* with LiESAp antigen killed promastigotes and amastigotes and inhibited the in vitro infectivity of promastigotes for canine macrophages [16]. Mice lacking IgG1 are more resistant to *L. mexicana* infection and mount a strong IgG2a/c antibody response [17]. *L. major* taken up via different phagocytic receptors produces different outcomes of infection: mice infected with IgG-opsonized parasites showed enhanced protective immunity as well as increased numbers of *L. major*–infected lesional dendritic cells, leading to production of IL-12 and priming of Th1 and Tc1 cells and efficient parasite killing by lesional macrophages [18]. C57BL/6 mice co-infected with *L. amazonensis* and *L. major* develop chronic disease and produce less antigen-specific antibodies compared to similarly co-infected C3HeB/FeJ mice, which heal [19]. These and other results indicate that the subclass, specificity, and, possibly, affinity of anti-*Leishmania* antibodies may determine outcome of infection.

Kedzierski and colleagues [20] caution against extrapolating results obtained with the murine models of immunity to humans: there are still no unambiguous correlates of protection, and the induction of a putatively protective IFN-γ response via vaccines will not be sufficient to induce protection if levels of IL-10 are disproportionately elevated. They propose that testing for IL-10 is as important as testing for IFN-γ to determine whether a vaccine has induced protective responses. So far the leishmanin (Montenegro) skin test is the most informative and practical immunological surrogate marker used in clinical trials [21]. With a complementary rationale Campos-Neto [22] has shown that disease-associated Th2 antigens of *Leishmania* can be protective if a Th1 response to them is generated before infection. Moreover, the immune response to a given antigen (e.g., LACK) can be protective (Th1) or exacerbating (Th2), depending on the way the antigen is introduced [23] or on the genetic background of the mouse. More recently, Nylén and Akuffo [24] proposed a strategy to search for the different biomarkers of outcomes of infection with *Leishmania* parasites in human subjects. These biomarkers may be essential not only for elucidating details of protective immunity, but for determining the efficacy of vaccines and for assisting in screening of novel antigens. This same approach is now also guiding research on AIDS vaccines [25]. The focus on individuals with infection but no clinical manifestations is logical because while natural immunity against *Leishmania* may not prevent infection, the fact that the majority of infections do not result in disease indicates that efficacious immune mechanisms exist. This situation is seen in dogs [26], and more emphasis should be given to canine immunology. This natural protective response should be the gold standard, and the development and efficacy of vaccines should be considered within the context of this standard.

### Persistence of Parasites: Role in Immunity Vaccine Development

Leishmanization is unacceptable for many individuals and regulatory agencies because it can be accompanied by minor complications and is potentially problematic in immunocompromised recipients; however, co-injection of live parasites with CpG oligodeoxynucleotides attenuates the severity of disease following leishmanization in mice [27]. Vaccination with live parasites that harbor suicide cassettes, making them susceptible to treatment with antibiotics, was tested successfully in mice as a means to provide a safe live challenge in clinical trial, but it has not been pursued further as a vaccine [28]. Leishmanization has not been tested against disease caused by species of *Leishmania* other than *L. major*; furthermore, the degree of cross-protection provided has not been sufficiently evaluated. It is noteworthy that epidemiological evidence indicates that individuals from Sudan with history of CL have lower incidence of VL [29]. Okwor and Uzonna [7] argue that vaccination with live-parasite-based vaccines for CL would induce effector and central memory cells. The challenge will be to achieve attenuation of live parasites without losing efficacy. In this regard, observations in animal models indicate that antigen persistence may be as important as the specific protein or parasite component employed in a vaccine [30].

### Vector Saliva and Parasite Proteo-Phosphoglycans: Role in Immunity and in Vaccine Development

Salivary antigens of the vector are important additional components of a vaccine [20]. This aspect has received little attention in spite of the fact that salivary proteins from the vector are also delivered to the host during natural transmission of the pathogen, and, in at least some cases, these proteins are immunogenic or immunomodulatory for the host [31], [32]. The rationales behind including salivary antigens of the sand fly vector in vaccines against the leishmaniases are 2-fold and are not necessarily mutually exclusive. If some of these salivary proteins are immunosuppressants, they may also compromise induction of protective immunity with *Leishmania*-derived antigens. Thus, a vaccine may neutralize this immunosuppressant activity. Conversely, several salivary proteins are potent immunogens and induce lymphocytic infiltration and production of IFN-γ and IL-12; this reaction may set the local stage for a protective anti-*Leishmania* immune response. Sand fly bites induce hypersensitivity reactions in many hosts, and the problem of how to target sand fly salivary proteins while avoiding these reactions also needs to be addressed. Rogers and colleagues [33] argue that parasite-secreted proteo-phosphoglycans, rather than vector saliva, are responsible for enhanced disease following sand-fly-transmitted versus needle infection and are viable vaccine targets. Proteo-phosphoglycans are regurgitated by phebotomines into the host, where they activate host macrophages through the alternative arginase pathway. Arginase synthesizes polyamines, which are essential growth factors for *Leishmania* [34]. The neglect of the role of the vector in vaccine development is reflected by the fact that the efficacy of all but two [35], [36] experimental vaccines has been tested by needle, rather than infected sand fly challenge (see the Table S1). Kedzierski and colleagues [20] call attention to the influence of wound repair triggered by the bite of the vector, a little-studied aspect of resistance to leishmaniasis.

## Antigens for Anti-Leishmaniasis Vaccines: State of the Art

Most of the 25 vaccines licensed for use in humans are effective because they drive responses to many different targets on the pathogens [37]. Therefore, the capacity to respond to multiple antigens may be an essential requisite of an efficacious vaccine. This observation brings up the issue of antigen discovery. As seen in Table S1, 34 subunits of *Leishmania* antigens are available, but they have not been evaluated as a set of more than three components. Given the complexity of the eukaryotic *Leishmania* parasite, the fact that the response observed following live vaccination or healed infection in humans is poly-specific and that immunization with a combination of the antigens LmST11 and TSA, a polyprotein vaccine, apparently confers the best degree of protection so far in nonhuman primates [38] suggests that an increase in the known repertoire of immunogenic antigens for this parasite is necessary. The new sequencing technologies should facilitate this task. Table S1 is a starting point, a database of vaccine candidates similar to that created for malaria [39]. A comparative analysis of the genomes of *Plasmodium vivax*, *P. falciparum*, *P. yoelii,* and *P. knowlesi* [40] revealed novel gene families and invasion pathways in *P. vivax*. A similar advance with more genomes of *Leishmania* parasites could reveal alternative mechanisms of parasitism and, thus, new targets for vaccines. Another case in point is *L. tarentolae*, a lizard pathogen that is nonpathogenic to humans. It expresses a cysteine protease B, lipophosphoglycan LPG3, and the leishmanolysin GP63, which are virulence genes of *Leishmania* species pathogenic for humans. On the other hand, the A2 gene, expressed by the *L. donovani* complex and which promotes visceralization, is absent in *L. tarentolae* [41]. *L. tarentolae* could play the same role in leishmaniasis control that vaccinia virus played in the eradication of smallpox, where a species nonpathogenic for humans induced protective immunity against a related species of pathogen. Finally, pathoantigens should be identified to better understand the pathological process, but also to preclude these molecules during selection of vaccine candidates and, if they are essential for protective responses, to research strategies to render them nonpathogenic.

**Figure 1:**
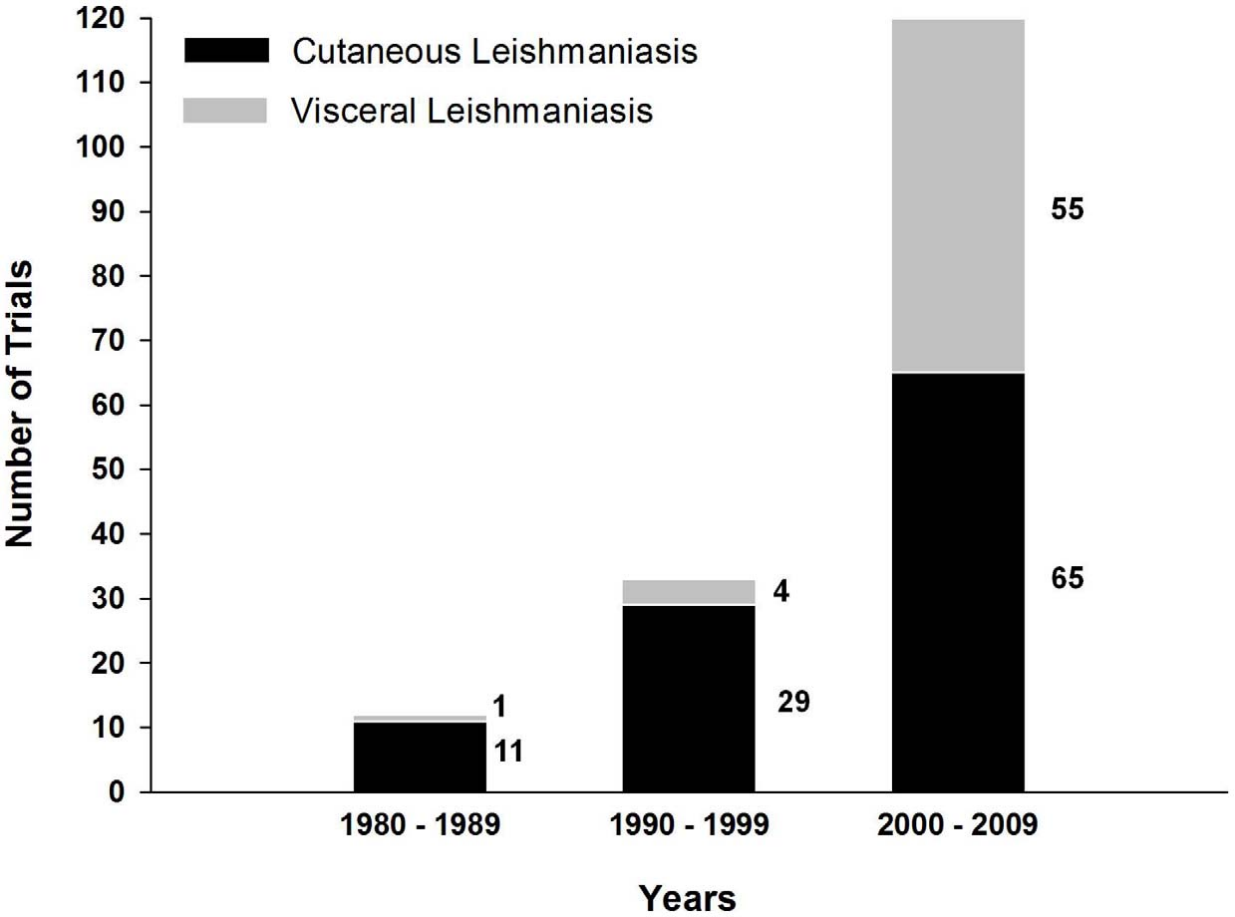
Number of *Leishmania* vaccine trials in last three decades. Data are derived from Table S1, which contains a summary of all vaccines to date (both experimental and in clinical use). Black bars represent CL: *L. major*, *L. mexicana*, *L. tropica*, *L. amazonensis*, and *L. braziliensis*; gray bars represent VL: *L. donovani*, *L. chagasi*, and *L. infantum*.

The number of experimental vaccines developed in the last 30 years that target the different forms of leishmaniasis surpasses 100. These experimental vaccines have undergone various stages of evaluation and comprise antigens consisting of whole live, attenuated, genetically modified, or killed parasites, or one or more subunits or fusion proteins. They employ different adjuvants, delivery systems, and vectors. Finally, they have been evaluated in five different animal models as well as in humans. Table S1 (an interactive version of this table is available at http://www.leishvaccines.net) and Figures 1–3 summarize all of these efforts, and the numbers they depict attest to the fact that vaccinology is still an imprecise science and can be bewildering to authorities and planners.

**Figure 2 pntd-0000943-g002:**
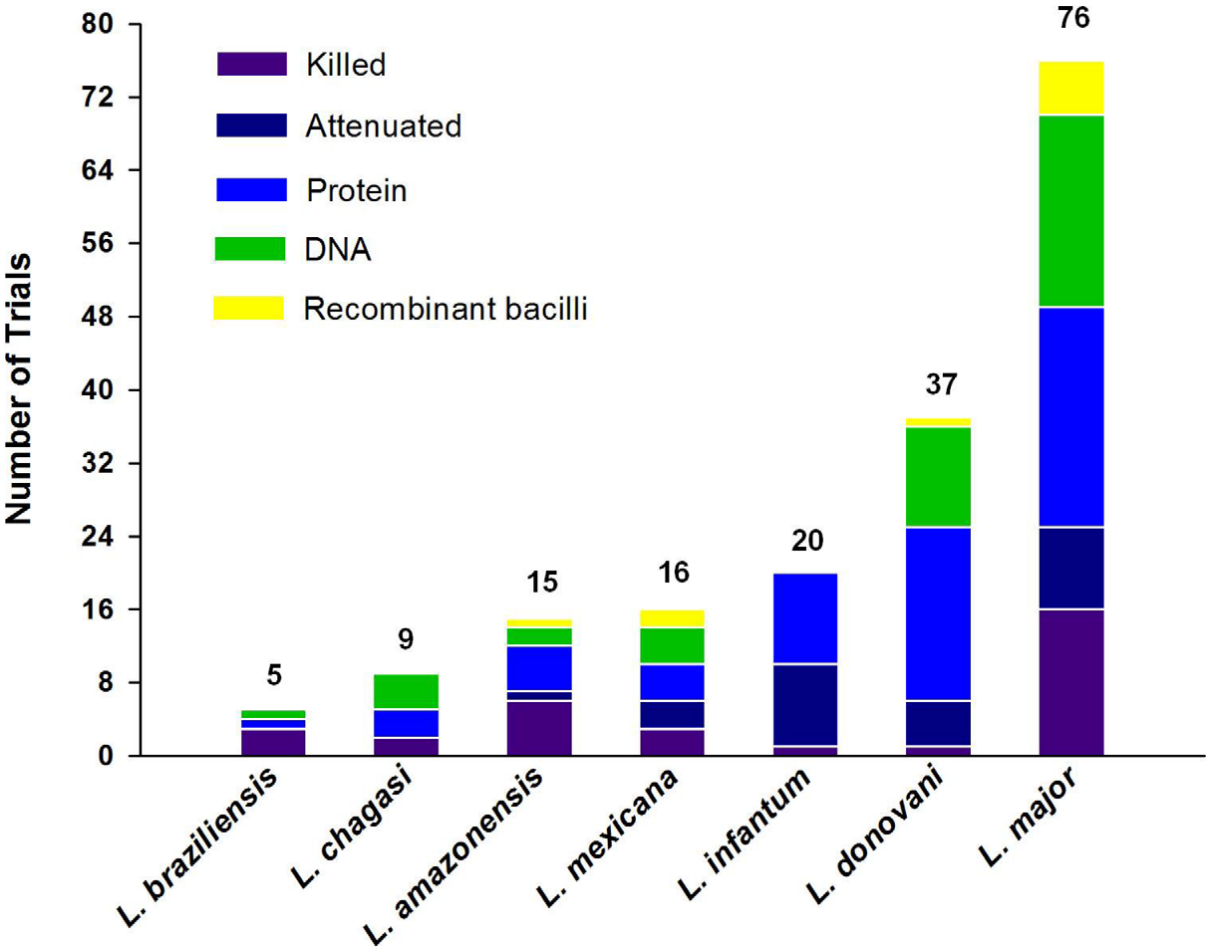
Profile of strategies used in leishmaniasis trials. Data are derived from Table S1, which contains a summary of all vaccines to date (both experimental and in clinical use). Bar graphs display the number of trials for each *Leishmania* species and the type of antigens that have been tested.

**Figure 3:**
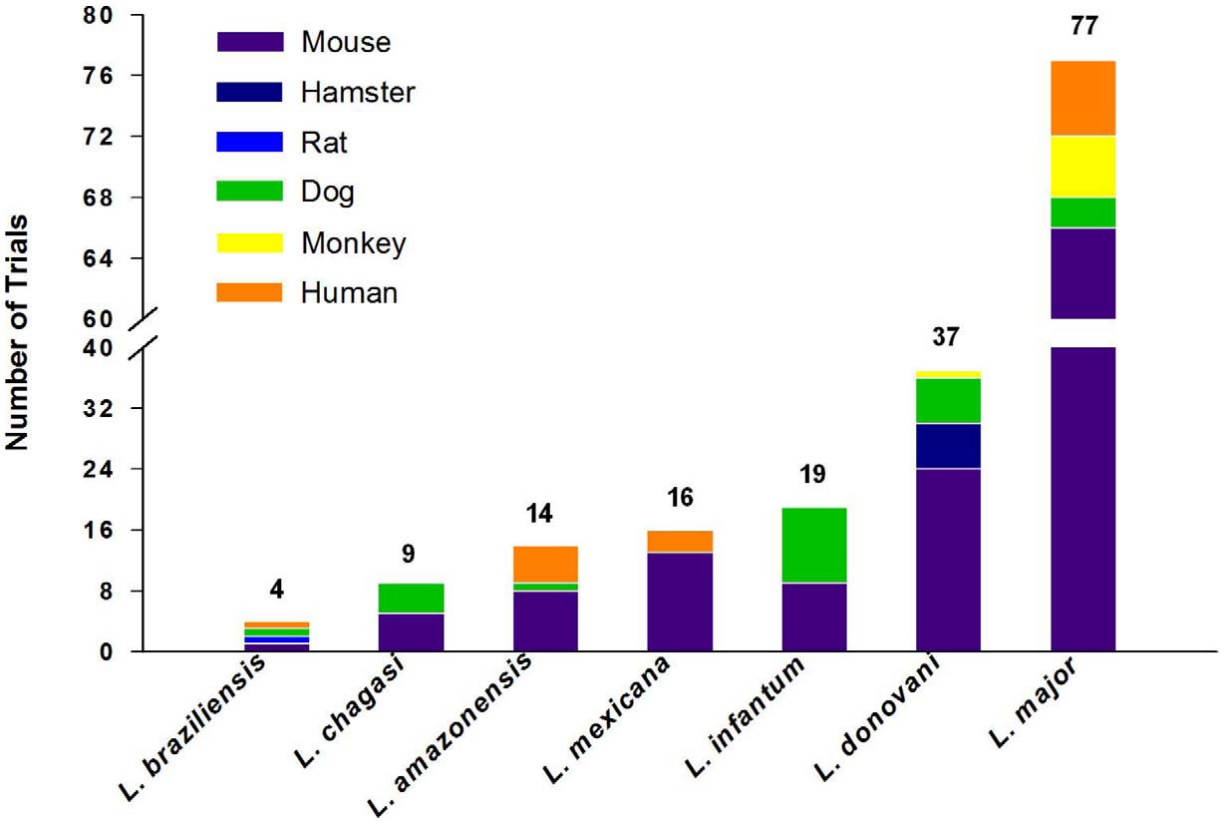
Animal models used in leishmaniasis trials. Data are derived from Table S1, which contains a summary of all vaccines to date (both experimental and in clinical use). Each color code reports the hosts in which *Leishmania* vaccines have been tested.

## Concluding Remarks

The main questions raised during debates at the International Symposium on Leishmaniasis Vaccines are summarized in Table 1. Except when specifically noted, the questions addressed issues concerning all of the leishmaniases. They addressed live attenuated or genetically modified (GM) parasites, the role of vectors, further antigen discovery, and elucidation of protective immunity for formulation of vaccines, models and clinical trials, funding policies, and dissemination of information on all these issues, on which the development of vaccines for the leishmaniases depends.

**Table 1:** Questions and issues raised at the International Symposium on Leishmaniasis Vaccines, and proposals for action through a research agenda.

**1. Vaccines That Employ Live Attenuated or GM Parasites or Antigens Delivered in the Form of Genetic (DNA) Vaccines**
• How long does infection with attenuated or GM organisms need to persist in order for vaccines to be effective and produce long-term immunity (before the infection is “cured” with drugs)?
• What kind of host response should be induced by a vaccine?
• Does *Leishmania* grown in chemically defined medium (serum-free due to safety concerns of vaccines made with cultured parasites) cause infection?
• How does the growth of live attenuated or GM parasites in axenic culture conditions impact their virulence?
• Why are GM parasites that lack virulence factors protective? What kind of response are they inducing, and what antigens are the vaccinated hosts recognizing?
• Do GM parasites, including knock-out parasites, over- or under-express proteins, including antigens of interest?
• What are the biomarkers of safety for live attenuated or GM parasites? How do we validate biomarkers of safety, and what would be the appropriate model to study that?
• Would leishmanization with innocuous species that express antigens of interest be protective?
• Safety and lack of toxicity of DNA vaccines and viral vectors are still unknown.
• Do we need adjuvants in combination of live attenuated or GM parasites as vaccine candidates? If so, what type, and when should they be used in the vaccination scheme?
• What is the impact of vaccines that employ live attenuated or GM parasites for immunocompromised individuals?
**2. The Role of Vectors and Vector Saliva in Modulating the Response to Vaccines and the Natural History of the Leishmaniases**
• Is anti-vector immunity priming specific anti-*Leishmania* immunity?
• Can different levels of exposure to vector saliva (seasonal variations, host attraction, and kairomones) affect the type of immune response to saliva and/or to the vector-borne pathogen?
• Can immunity to vectors have a herd effect on disease by affecting transmission and/or viability of vectors?
• What constitutes efficacy?
• More knowledge is needed on the natural history of the leishmaniases, especially if integrated control is necessary because of the lack of a vaccine with 100% efficacy or difficulties in achieving 100% coverage.
• Further quantitative epidemiology studies and mathematical models, to predict the community effects of vaccination using a vaccine with <100% efficacy or <100% coverage, are needed.
• Vaccines should be evaluated by insect challenge.
• Should vaccines with attenuated live parasites be transmitted by the vector in order to confer herd immunity?
**3. Antigen Discovery for Formulation of Vaccines**
• What are the determinants of diverse biological behavior/characteristics of parasites of the same species?
• Considering that lesions in human tegumentary leishmaniasis (except diffuse CL) are the result of hypersensitivity reactions, the identification of pathoantigens is necessary to understand the pathological process and preclude these molecules during antigen selection.
• Do we need more antigens (we already have 34 subunits; see Table S1)?
• What are good tools and strategies for discovering novel antigens?
• Should reverse vaccinology continue to be exploited?
• What are the criteria for defining antigens of interest when employing reverse vaccinology?
• Is the comparison of genomes of pathogens with those of nonpathogenic species useful?
• Are studies with single nucleotide polymorphisms on genetic variability/antigenic variation within host and parasite useful? Genotyping chips for humans and dogs are available; a genotyping chip should be generated for *Leishmania* sp.
• Why are the antigens under evaluation giving insufficient protection? What is lacking in the host's response? Do we need more adequate adjuvants for them? Do we need more vaccination protocols, such as prime-boosting strategies? Are the antigens able to induce long-lasting protection?
• Why are some parasitic proteins immunogenic, while others are not? Can non-immunogenic proteins, as defined by patient sera, be protective antigens?
• What are the best delivery systems and regimens?
• What are the parasitic proteins that cause pathology? Should we focus on them for use as antigens?
• Antigens already under study should be better characterized concerning broadness of immunogenetic restriction, density and type of epitopes, role in parasite's biology, etc.
• More knowledge is needed about the species that cause mucocutaneous leishmaniasis and diffuse CL and the immune responses involved in these clinical outcomes.
• More knowledge is needed about the commonalities between the different causative agents of CL to explain why the different infections cause similar clinical presentations.
**4. Immune Responses of Natural Hosts and Models**
• It is crucial to test vaccine candidates in different models using different species, and to test the effects of including salivary proteins in vaccines.
• Because of the difficulty of finding a good model of human leishmaniasis, before human trials, the nonhuman primate model appears to be an important option to test candidate vaccines.
• What is the significance of subclinical infections? Are they useful for determining mechanisms of protection?
• DNA banks and genome-wide association studies with single nucleotide polymorphism chips can assist in determining the mechanisms behind the different outcomes of clinical and subclinical infections with *Leishmania*; likewise whole-genome comparative expression profiling can dissect mechanisms of resistance and susceptibility.
• Will T cells and antibodies from healthy hosts presenting with subclinical infections with *Leishmania* recognize different antigens/epitopes during expression cloning for antigen discovery than T cells and antibodies from patients presenting clinically manifest disease? Are these antigens more useful to formulate vaccines?
• What kind of immune response is protective? What are the surrogate markers of protection?
• Is it important to avoid Th2/T regulatory–type responses to *Leishmania* and not just induce Th1/IFN-γ responses?
• Are antibody responses part of a protective response?
• If antibodies are an important effector mechanism to be elicited by vaccines, how will current regulatory issues on vaccines for dogs be addressed?
• Do antibodies/immune responses exert selective pressure on antigens to undergo antigenic variation? Which antigens are they, and what is their role in the parasite's biology? Is it an important role?
• What is the role of antigen processing in mounting protective immune responses?
• What is the role of neutrophils in protection from or susceptibility to *Leishmania*?
• What is the role of co-infections and nutritional status in the immune responses to *Leishmania* and to vaccines?
• What is the role of the host's genetic background in susceptibility to clinical manifestations of infections with *Leishmania* and responses to vaccines?
**5. Models and Clinical Trials**
• Uniform challenges in both models and humans need to be implemented to test the different antigens in a comparative fashion.
• Again, what constitutes protection/efficacy?
• What are the surrogate markers of protection, and how are they measured?
• Crude *Leishmania* preparations for leishmanin (Montenegro) skin tests and first generation vaccines are subject to batch-to-batch variation, an issue which needs to be urgently addressed.
**6. Funding for Vaccine Development**
• From the points mentioned herein, it is important to recommend funding for primate facilities.
• Funding for access to good manufacturing processes facilities and for sand fly experiments in vaccine development is also important.
**7. Sharing Information**
• The LeishNet Web site (http://leishnet.net) should be reissued. A Leish-L list of E-mails linked to more modern tools as well as to an area specific for leishmaniasis vaccines, with an interactive approach similar to Wikipedia, should be constructed.

Funding agencies must be persuaded that the profile of the leishmaniases is shifting and that a concerted action is needed with new scientific approaches. A strategic plan is needed to attain truly efficacious vaccines against the leishmaniases. The first bottleneck to overcome is caused by scientific gaps; therefore, a call for grant proposals should focus on a few scientific questions and research priorities in vaccinology for VL and CL that are potentially very informative. Proposals should ideally be executed as a network in order to maximize existing information generated by individual groups and to generate new insights. The research priorities could be validated through consultations with participants in the discussion group that the Working Group has set up (http://groups.google.com.br/group/leishvaccines-l) during a fixed period of time, six months. In principle these research priorities might address the following:

- The role of parasite persistence in protective immunity and live versus killed/subunit vaccines in order to define (i) a product profile for VL and CL and (ii) whether it is valid to improve leishmanization for CL;
- The role of immunity to vector saliva in immunity against different species of *Leishmania* and in composition of vaccines;
- The significance and immunological profile of the hosts who are able to maintain subclinical infections and their role in obtaining biomarkers of resistance and protective immunity;
- Further application of genomic approaches for (i) identifying virulence factors in different species of *Leishmania* and (ii) antigen discovery;
- Re-formulation of a previously tested subunit vaccines that include novel antigens, including antigens of the 30 subunits previously identified by different groups (Table S1), and/or new adjuvants.

Conceptual and technological advances in immunology, parasitology, genetics, genomics, and bioinformatics have increased our capacity to address the research priorities with a more effective systemic approach to the vector–*Leishmania*–host interface. However, these advances do not affect the constraints that spring from the placement of political will and personal scientific convictions. The size of this task and the seriousness of the problem represented by these neglected diseases warrant a large collaborative effort. At least 30 vaccine subunits (listed in the Table S1) have been tested with relative success by several different groups. If a multicomponent vaccine is a sine qua non condition for achieving an effective vaccine, many of these antigens will probably be part of the composition. In this case, the issue of intellectual property becomes an significant barrier for attracting companies willing to develop, manufacture, and market a vaccine. The funding agencies and grantees must devise a solution that is fair for all interested parties, including the patients.

An example in science where competing groups (with large financial interests at stake) eventually united and then achieved a common goal is seen in the cloning of the gene causing cystic fibrosis. In contrast to the the situation for patients with neglected leishmaniases, patients with cystic fibrosis and their families count on the active Cystic Fibrosis Foundation, incorporated in 1955, which created a network of research centers and a research program in 1980, the same time that efforts began for developing subunit vaccines for *Leishmania*. In less than nine years, in the pre-genomics era, the defective cystic fibrosis gene and its protein product were described. Presently, more than 30 drugs that modulate the cystic fibrosis gene product itself are in the pipeline, including phase 3 trials. Individuals at risk for developing leishmaniasis need a similar voice and concerted action.

## Supporting Information

Table S1 A survey of antigens, adjuvants, delivery systems, and models employed for developing vaccines for the VLs and CLs.
(0.99 MB DOC)
