# Supplementary material for: Vaccines for the Leishmaniases: Proposals for a Research Agenda
Source: PLoS Negl Trop Dis. 2011 Mar 29;5(3):e943. doi: 10.1371/journal.pntd.0000943 (PMC3066138; doi:10.1371/journal.pntd.0000943)
Supplement: Table S1 — A survey of antigens, adjuvants, delivery systems, and models employed for developing vaccines for the VLs and CLs. (0.99 MB DOC) [file pntd.0000943.s001.doc]

Supplemental Table. A survey of antigens, adjuvants, delivery systems and models employed for developing vaccines for the visceral and cutaneous leishmaniases.

| **Species of Leishmania**  **Causing disease**  **Targeted by vaccine** | **Species of Leishmania**  **From which antigen component of vaccine is derived^1^** | **Type of antigen**  Live  Live, Attenuated  Killed  Recombinant bacilli  Recombinant virus  Subunit  -Number of subunits/fusion | **Form of delivery**  Protein  DNA /Vector  Recombinant bacilli | **Route of vaccine delivery**  SC  IM  Mucosal  IP  ID  IV | **Regimen** | **Challenge**  Needle  Vector  -Natural  -Experimental | **Host/Model**  Primate  -Human (Phase)  -Monkey  Mouse  Hamster  Dog (Phase) | **Adjuvant** | **Effector mechanism elicited** | **Protection**  - No  - Partial  - Strong  - Not evaluated | **Ref** |
| --- | --- | --- | --- | --- | --- | --- | --- | --- | --- | --- | --- |
| L. infantum | 1. *L. infantum* | Subnit:  KMP-II, TRYP, LACK, GP63 | DNA/pMOK | ID | 200 μg of each plasmid DNA constructs. | Needle | Dog (Phase N/A*) | - | ↑ IgG and IgA (No protection) | No Protection | [1] |
|  | 2. *L. infantum* | Subnit:  LACK | DNA/pCI-neo | SC | 100 μg of plasmid DNA construct. Boost 15 days after with 10^8^ pfu of vaccinia virus expressing LACK | Needle | Dog (Phase N/A*) | - | Th1 response | Partial protection | [2] |
|  | 3. *L. infantum* | Subnit:  CPB and CPA | Protein DNA/pCB6 | IM | 100 μg of each plasmid DNA constructs. Boost 1 month (DNA) and 2 month after (protein) | Needle | Dog (Phase N/A*) | CpG ODN and Montanide 720 | Th1 response  (IFN-γ mRNA and ↑ IgG2) | Strong protection | [3] |
|  | 4. *L. infantum* | Subunit:  CPB and CPA | DNA/pCB6 | SC | 100 μg of each plasmid DNA constructs. Boost 1 month (DNA) and 2 month after (protein) | Needle | Mouse (Balb/c) | CpG ODN and Montanide 720 | Th1 response  (IFN-γ and ↑ IgG2) | Partial protection | [4] |
|  | 5. *L. infantum* | Subunit:  CTE | Protein and DNA/pCDNA3 | SC | 100 μg plasmid DNA construct. Boost 3 wk later with recombinant protein CTE | Needle | Mouse (Balb/c) | CpG motif and Montanide 720 | Th1 and Th2 responses | No Protection | [5] |
|  | 6. *L. major* | Subunit:  LeIF, LmSTI1 and TSA | Protein | SC | 10 μg of the Leish-111 f ( polyprotein containing LmSTI1 and LeIF fusioned), three times, 3 wk apart | Needle | Mouse (Balb/c) | MPL-SE | Th1 response | Partial protection | [6] |
|  | 7. *L. major* | Subunit:  LeIF, LmSTI1 and TSA | Protein | SC | 45 μg of the Leish-111 f ( polyprotein containing LmSTI1 and LeIF fusioned), three times, 3 wk apart | Needle | Dog (Phase III) | MPL-SE and AdjuPrime | Th1 response | No Protection | [7] |
|  | 8. *L. infantum* | Subunit:  H2A, H2B, H3 and H4 | DNA/pCDNA3 | IM | 80 μg of each of plasmid DNA construct, three times, 2 wk interval. Alternatively, was a DC-based vaccine (dendritic cells was pulsed with different histones and transferred to mice) | Needle | Mouse (Balb/c) | - | Th1 response | No Protection | [8] |
|  | 9. *L. infantum* | Subunit:  LiESAp (purified excreted-secreted antigens) | Protein | SC | 100 μg of protein. Boost 3 wk later. | Needle | Dog (Phase I) | MDP | Th1 response  (IgG2) | Strong protection | [9] |
|  | 10. *L. infantum* | Subunit:  CPC (cysteine proteinase type  III) | DNA/pcDNA | SC | 100 μg plasmid DNA construct. Boost 3 wk later with 30 μg recombinant protein. | Needle | Mouse (Balb/c) | Montanide and CpG motif | Th1 response | Strong protection | [10] |
|  | 11. *L. infantum* | Subunit:  LACK | DNA/pCI-neo | SC | 100 μg plasmid DNA construct. Boost with 10^7^ pfu of recombinant virus (VV or MVA) expressing LACK 15 and 30 days later. | Needle | Dog (Phase I) | - | Th1 response | Strong protection | [11] |
|  | 12. *L. infantum* | Dendritic cells pulsed with histones (H2A, H2B, H3 and H4) |  | IV | 10^6^ cells | Needle | Mouse (Balb/c) | - | Th1 response | Partial protection | [12] |
|  | 13. *L. infantum* | Subunit:  LiESAp (purified excreted-secreted antigens) | Protein | SC | 100 μg of protein. Boost 4 wk later. | Needle | Dog (Phase I) | MDP | Th1 response  (IgG2) | Partial protection | [13] |
|  | 14. *L. infantum* | Subunit:  CPA and CPB (cysteine peptidases) | Protein | SC | 50 μg of each protein, combined. Boost 4 wk later. | Needle | Dog (Phase I) | Quil A and IL-12 exogenous | Th1 response | No Protection | [14] |
|  | 15. *L. infantum* | Subunit:  LACK | DNA/pCI-neo | ID | 100 μg of plasmid DNA construct. Boost 2 wk later with 10^7^ pfu of recombinant vaccinia viruses expressing LACK antigen | Needle | Mouse (Balb/c) | - | Th1 response | Partial protection | [15] |
|  | 16. *L. infantum* | Subunit:  LiESAp | Protein | SC | 50, 100 or 200 μg of protein. Boost 3 wk later. | Needle | Dog (Phase I) | MDP (muramyl dipeptide) | Th1 response | Partial protection | [16] |
|  | 17. *L. infantum* | Subunit:  Poly-protein Q (containing five genetically  fused antigenic determinants from the Lip2a, Lip2b, H2A and  P0 proteins) | Protein | IP | 2 μg of polyprotein Q. Boost 2 wk later. | Needle | Mouse (Balb/c) | BCG, CPG or Freund’s adjuvants (CFA and IFA) | Th1 response | Partial protection | [17] |
|  | 18. *L. infantum* | Killed |  | SC | 3 x 10^7^ promastigotes, single dose. | - | Dog (Phase I) | CFA | Th1 response | Not Evaluated | [18] |
|  | 19. *L. infantum* | Subunit:  LiHsp83 (heat-shock protein) fusioned with MBP | Protein | IP | 3 μg of fusioned protein, 2 doses with 3 wk interval. | Mice (CF1) | Mice (CF1) | - | Th1 response | Not Evaluated | [19] |
| *L. donovani* | 1. *L. donovani* | Subunit:  Kinetoplastid Membrane Protein-II (KMP-11) | DNA/pCMV-LIC | IM | 100 μg of plasmid DNA constructs,  7 and 15 days before the challenge with LD. | Needle | Mouse (Balb/c) | +/- exogenous IL-12 (subcloned from pED vector in pCMV-LIC); SC route | mixed Th1/Th2 response (KMP-11 alone) | Partial Protection | [20] |
|  | 2. *L. donovani* | Subunit:  Kinetoplastid Membrane Protein-11 (KMP-11) | DNA/pCMV-LIC | IM | 100 μg of plasmid DNA constructs,  7 and 15 days before the challenge with AG83 and GE1F8R  strains of *L. donovani.* | Needle | Hamster | - | CTL-like response; mixed Th1/Th2 cytokine response | Strong protection | [21] |
|  | 3. *L. major* | Recombinant bacilli:  *Salmonella* expressing Gp63 | Recombinant bacilli | Mucosal (oral) | 5 x 10^9^ recombinant bacilli. Single oral dose | Needle | Mouse (Balb/c) | - | Th1 response (IFN-γ) | Partial protection | [22] |
|  | 4. *L. donovani* | Subunit:  LACK | DNA/pCDNA3 | ID (ear and rump) SC (tail and footpad) | 100 μg of plasmid DNA construct. | Needle | Mouse (Balb/c) | - | Th1 response (IFN-γ) | No protection | [23] |
|  | 5. *L. donovani* | Subunit:  Lag (promastigote  membrane antigens) | Protein | IP and IV | 20 μg of proteins. Boost 2 wk and 4 wk later. | Needle | Mouse (Balb/c) | Liposome | Th1 response | Strong protection | [24] |
|  | 6. *L. donovani* | Subunit:  FML (purified fraction of fucose mannose ligand) | Protein | SC | 1.5 mg of protein 3 doses, 3 wk intervals | - | Dog | *Quillaja saponaria* saponins | Mixed Th1/Th2 response | Not Evaluated | [25] |
|  | 7*. L. amazonensis* | Live, suicidal mutant (transgenic *L. Amazonensis*) | - | ID | 10^7^ Transgenic DT Leishmania and ALA 3 days later | Needle | Hamster | - | Th1 response | Strong Protection | [26] |
|  | 8. *L. donovani* | Live, attenuated (UDP-Gal) | - | SC | 10^7^ A-LD parasites twice at 15 wk interval | Needle | Mouse (Balb/c) | - | Th1 and DTH responses | Partial Protection | [27] |
|  | 9. *L. donovani* | Subunit:  F14 (ribosomal protein P0) and A6 | Protein | IM | 50 μg of proteins, alone or together, 3 doses at 2-  wk interval | Needle | Hamster | MPL | Th1 response | Partial Protection | [28] |
|  | 10. *L. donovani* | Subunit:  Lvacc  (kinesin protein from the microtubule) | DNA/pVAX-1 | IM | 100 μg of plasmid DNA construct. Boost 2 wk later. | - | Mouse (Balb/c) | - | Th1 response | Not Ealuated | [29] |
|  | 11.  *L. donovani* | Subunit:  Gp63 | Protein (purified Soluble leishmanial antigens) | IP | 10 μg of protein. Boost 2 wk later. | Needle | Mouse (Balb/c) | DSPC liposomes | Th1 response | Partial Protection | [30] |
|  | 12.  *L. donovani* | Subunit:  SLA (soluble leishmanial  Antigens) | Protein | IP | 15 μg of protein. Boost 2 ansd 4 wk later. | Needle | Mouse (Balb/c) | Liposomes | Th1 response | Strong Portection | [31] |
|  | 13. *L. donovani* | Subunit:  γ-GCS (gamma-glutamylcysteine synthetase) | DNA/pVAX | IM | 50 μg of plasmid DNA construct. Boost 2 and 4 wk later. | Needle | Mouse (Balb/c) | - | Humoral response (IgG1 and IgG2) | Partial Protection | [32] |
|  | 14. *L. donovani* | Subunit:  FML-Leishimune | Protein | SC | Three vaccine doses, with 20-  day intervals |  | Dog | Saponin | Humoral response inhibited Lutzomyia longipalpis infection in vivo and inhibits the L. donovani and L.  chagasi binding to sand fly midguts | Partial Protection | [33] |
|  | 15. *L. donovani* | Subunit:  H2A, H2B, H3, H4 and LACK | DNA/pCDNA3.1 | IM | 100 μg of each plasmid DNA constructs, together (500 μg total). Boost 30 and 60 days later. | Needle | Dog | - | Th1 response | Partial Protection | [34] |
|  | 16. *Leishmania tarentolae* | Live, nonpathogenic to humans |  | IP | 5 x 10^6^  stationary-phase *L. tarentolae* promastigotes | Needle | Mouse (Balb/c) | - | Th1 response | Partial Protection | [35] |
|  | 17. *L. donovani* | Subunit:  FML-Leishimune | Protein | SC | 3 doses with 3 wk interval. | - | Dog | Saponin | Not evaluated | Strong Protection | [36] |
|  | 18. *L. donovani* | Subunit:  ORFF (open reading frame) form LD1 locus | DNA/pCDNA3.1 | IM | 100 μg of plasmid DNA construct. Boost 2wk later with 50 μg of recombinant protein | Needle | Mouse (Balb/c) | Alum | Th1 response | Partial Protection | [37] |
|  | 19. *L. donovani* | Subunit:  FML-Leishimune | Protein | SC | 150 μg of protein, 3 doses weekly. | Needle | Mouse (Balb/c) | CP05 (saponin from leaves of *Calliandra pulcherrima*) | DTH and Humoral response | Not Evaluated | [38] |
|  | 20. *L. donovani* | Subunit:  SLA (soluble leishmanial  Antigens) | Protein | IM | 50 μg of protein. Boost 2 wk later. | Needle | Mouse (Balb/c) | CPG-ODN | Th1 response | Partial Protection | [39] |
|  | 21. *L. donovani* | Subunit:  LAg | Protein | IP | 20 μg of protein. Boost 2 and 4 wk later. | Needle | Mouse (Balb/c) | Liposome | Mixed Th1/Th2 response | Strong Protection | [40] |
|  | 22. *L. donovani* | Subunit:  LPG (lipophosphoglycan) |  | IP (Mouse) or SC (Hamster) | 10 μg (Mouse) or 20 μg (Hamster) of LPG, 3 doses with 2 wk interval. | Needle | Hamster and Mouse (Balb/c) | BCG | Th1 response | No protection | [41] |
|  | 23. *L. donovani* | Subunit:  ORFF (open reading frame) form LD1 locus | DNA/pCDNA3.1 | IM | 100 μg of plasmid DNA construct. 3 doses with 3 wk interval. | Needle | Mouse (Balb/c) | - | Th1 response | Partial Protection | [42] |
|  | 24. *L. donovani* | Subunit:  FML | Protein | SC | 1,5 mg of protein, 3 doses with 3 wk interval. | - | Dog (Phase III) | QuilA saponin | Humoral response | Strong Protection | [43] |
|  | 25. *L. donovani* | Live, attenuated (bioterin transporter - BT1 null mutant) |  | IV | 1 x 10^7^ of mutant promastigotes | Needle | Mouse (Balb/c) | - | Th1 response | Partial Protection | [44] |
|  | 26. *L. donovani* | Subunit:  FML | Protein | SC | 1,5 mg of protein, 3 doses with 3 wk interval. | - | Dog (Phase III) | - | DTH and Humoral response were evaluated | Strong Protection | [45] |
|  | 27. *L. donovani* | Subunit:  rHASPB1 (recombinante hydrophilic acylated surface protein  B1 ) | Protein | SC | 10 μg of protein, , 3 doses with 3 wk interval.. | Needle | Mouse (Balb/c) | - | Th1 response | Partial Protection | [46] |
|  | 28. *L. donovani* | Subunit:  FML | Protein | SC or IP | 150 μg of protein, 3 weekly doses. | Needle | Mouse (Swiss Albino) | Saponin, CFA, IFA and aluminum hydoxide | Humoral response | Partial Protection | [47] |
|  | 29. *L. major* | Killed (ALM) | *Leishmania* proteins |  | 3 mg of protein, single dose. | Needle | Primate - Monkey | BCG | Not evaluated | Partial Protection | [48] |
|  | 30. *L. donovani* | Subunit:  FML | Protein | IP | 3 doses weekly | Needle | Mouse (Balb/c) | Saponin | Humoral response | Partial Protection | [49] |
|  | 31. *L. donovani* | Subunit:  dp72  and gp70-2 | Protein (purified from crude extract) | IP | 10 μg of protein, alone or together. Boost 25 days later with 2.5 μg of protein. | Needle | Mouse (Balb/c) | *Corynebacterium paruum* | No significant protection | gp70 - No protection dp72 - Partial protection | [50] |
|  | 32 *L. donovani* | Subunit:  Microsomal fraction | Protein | IP | 200 μg of protein, 4 doses at 4 aus interval. | Needle | Hamster | Beta 1,3 glucan | DTH and Humoral response | Partial Portection | [51] |
|  | 33. *L. donovani* | Live, Attenuated (centrin gene-deleted null mutant) | - | IV | 3 x 10^6^ parasites | Needle | Mouse (Balb/c-SCID) | - | Th1 response, IgG2a, Nitric oxide production. | Partial Portection | [52] |
|  |  |  |  | intracardial | 10 x 10^6^ parasites | Needle | Hamster |  | Reduced parasite burden |  |  |
|  | 34. *L. donovani* | Subunit: four proteins purified from promastigotes (LD91, LD72, LD51 and LD31) | Protein | IP | 2.5 μg. Boost twice with 2 wk interval | Needle | Mouse (Balb/c) | Cationic liposomes | Th1 response | Mixed response, depending on the polypeptides | [53] |
| *L. chagasi* | 1. *L. chagasi* | Subunit:  LACK | DNA/pCI-neo | SC (hind footpad) and IM (hind leg thigh) | 100 μg of plasmid DNA construct. | Needle | Mouse (Balb/c) | - | Th1 response (IFN-γ) | No protection | [54] |
|  | 2. *L. infantum* | Subunit:  LACK | DNA/pCI-neo | Mucosal (nasal) | 15 μg of plasmid DNA construct. Boost 1 wk after. | Needle | Mouse (Balb/c) |  | Th1 response (IFN-γ) | Strong protection | [55] |
|  | 3. *L. major* | Subunit:  LeIF, LmSTI1 and TSA | Protein | SC | 10 μg of the Leish-111 f ( polyprotein containing LmSTI1 and LeIF fusioned), three times, 3 wk apart | Needle | Dog (Phase I) | MPL-SE and AdjuPrime | Th1 response | Strong protection | [56] |
|  | 4. *L. donovani* | Subunit:  A2 | Protein | SC | 100 μg of recombinant protein. Boost 3 and 6 wk later. | Needle | Dog (Phase I) | Saponin | Th1 response | Partial protection | [57] |
|  | 5. *L amazonensis* | Killed (three rounds of  freezing and thawing) |  | SC | 100 μg of extract. Boost 2 wk later. | Needle | Mouse (Balb/c) | *Corynebacterium parvum* | Mixed Th1/Th2 responses | Strong protection | [58] |
|  | 6. *L. donovani* | Subunit:  NH and A2 | DNA/ A2-pCDNA3 and NH-VR1012 | IM | 100 μg of plasmid DNA constructs. Boost 3 and 6 wk later. | Needle | Mouse (Balb/c) | - | Th1 response | Partial protection | [59] |
|  | 7. *L. donovani* | Subunit:  FML | Protein | SC | 150 μg of protein, three weekly doses | Needle | Mouse (Swiss) | Saponin | Th1 response | Strong protection | [60] |
|  | 8. *L. donovani* | Subunit:  H2A, H2B, H3, H4 and LACK | DNA/pCDNA3.1 | IM | 100 μg of each plasmid DNA constructs, together (500 μg total). Boost 30 and 60 days later. | Needle | Dog | - | Th1 response | Partial protection | [34] |
|  | 9. *L. braziliensis* | Killed | Protein (crude extract) |  | 600 μg of protein |  | Dog (Phase I and II) | BCG |  | Partial protection | [61] |
| *L. major* | 1. *L. donovani* | Subunit:  Kinetoplastid Membrane Protein-11 (KMP-11) | DNA/pCMV-LIC | IM | 100 μg of plasmid DNA constructs,  7 and 15 days before the challenge with LM. | Needle | Mouse (Balb/c) | +/-exogenous IL-12 (subcloned from pED vector in pCMV-LIC); SC route | high IgG2a titre - Th1 response (KMP-11 + IL-12); | Partial Protection | [20] |
|  | 2. *L. major* | Subunit:  26 syntentic 9-10 mer peptides. 14 peptides have resulted to be immunogenic | Peptide | SC | 250 μg of pools of five peptides. Boost 2-3 wk after the 1^st^ immunization. | None - mice were sacrified and spleen removed. | Mouse (Balb/c) | 1^st^  dose CFA  2^nd^ dose IFA | T cell IFN-γ production | Partial Protection | [62] |
|  | 3. *L. major* | Subunit:  Native glycoconjugate (Gp63) isolated from parasite culture supernatant | Protein | IP | 15 μg of native protein/dose; 3 immunizations | Needle | Mouse (Balb/c) | *Corynebacterium parvum*  or CFA | Not evaluated (protection was analyzed by lesion score) | No Protection | [63] |
|  | 4. *L. major* | Subunit:  Syntetic peptide (PT3 epitope) from Gp63 | Peptide | SC |  |  | Mouse (Balb/c) | poloxamer 407 |  | Strong Protection | [64] |
|  | 5. *L. major* | Subunit:  cDNA Gp63 cloned in plasmid vector | DNA/pCDNA3 | ID | 100 μg of plasmid DNA construct. Boost 3 wk after 1^st^ immunization. | Needle | Mouse (Balb/c) | - | Th1 response (IFN-γ) | Partial Protection | [65] |
|  | 6. *L. major* | Recombinant bacilli:  BCG expressing Gp63 | Recombinant bacilli |  |  |  | Mouse (Balb/c) |  |  | Strong Protection | [66] |
|  | 7. *L. major* | Recombinant bacilli:  BCG expressing Gp63 cloned in pMV262 | Recombinant bacilli | SC | 10^6^ recombinant bacilli | Needle | Mouse (Balb/c) | - | Not evaluated (protection was analyzed by lesion score) | Partial Protection | [67] |
|  | 8. *L. major* | Recombinant bacilli:  *Salmonella* expressing Gp63 | Recombinant bacilli | Mucosal (oral) | 5 x 10^9^ recombinant bacilli. Boost after 2 wk. | Needle | Mouse (CBA) | - | Th1 response (IFN-γ) | Strong Protection | [68] |
|  | 9. *L. major* | Recombinant bacilli:  *Salmonella* expressing Gp63 | Recombinant bacilli | Mucosal (oral) | 10^6^ recombinant bacilli. Boost after 2 wk. | Needle | Mouse (Balb/c) | - | Th1 response (IFN-γ) | Strong Protection | [69] |
|  | 10. *L. major* | Recombinant bacilli:  *Salmonella* expressing Gp63 | Recombinant bacilli | Mucosal (oral) | 5 x 10^9^ recombinant bacilli. Single oral dose | Needle | Mouse (Balb/c) | - | Th1 response (IFN-γ) | Strong Protection | [22] |
|  | 11. *L. major* | Subunit:  Recombinant Gp63 protein fusioned with GST | Protein | ? | ? | Needle | Mouse (Balb/c) |  | No protection | No protection | [70] |
|  | 12. *L. major* | Subunit:  Gp63 | DNA/pCMV | IM | 100 μg of plasmid DNA constructs. Single dose. | Needle | Mouse (Balb/c) | - | Th1 response (IFN-γ) | Strong Protection | [71] |
|  | 13. *L. major* | Subunit:  Gp63, LACK, PSA2, LeIF, p20, Ribossomal-like | DNA/ pCMV3ISS | IM | 100 μg of plasmid DNA constructs. Single dose. | Needle | Mouse (Balb/c) | - | Not evaluated (protection was analyzed by footpad swelling) | Partial Protection | [72] |
|  | 14. *L. major* | Subunit:  LACK | Protein |  |  |  |  | exogenous IL-12 |  | Partial Protection | [73] |
|  | 15. *L. major* | Subunit:  LACK | DNA/pCDNA3 | IM | 100 μg of plasmid DNA constructs. Single dose. | Needle | Mouse (Balb/c) | exogenous IL-12 (cloned in pCDNA-3) or rIL-12 | Th1 response (IFN-γ) | Partial Protection | [74] |
|  | 16. *L. major* | Recombinant bacilli:  *Listeria* expressing LACK | Recombinant bacilli | IV | 5 x 10^7^ recombinant bacilli. Boost 2 wk after. | Needle | Mouse (Balb /c and B10.D2/  NOIaHsd) | - | CD8^+^ response; Th1 response | No protection | [75] |
|  | 17. *L. infantum* | Subunit:  LACK | DNA/ pCDNA3.1 | ID | 100 μg of plasmid DNA constructs. Boost 2 wk after with 10^7^ pfu of vaccinia virus expressing p36/LACK | Needle | Mouse (Balb/c) | - | Th1 response (IFN-γ) | Partial Protection | [76] |
|  | 18. *L. infantum* | Subunit:  LACK | DNA/ pCDNA3.1 | ID | 100 μg of plasmid DNA constructs. Boost 2 wk after with 10^7^ pfu of vaccinia virus expressing p36/LACK | Needle | Mouse (Balb/c) | IL-12 and IL-18 cloned in | Th1 response (IFN-γ) | Strong Protection | [77] |
|  | 19. *L. major* | Subunit:  LACK | DNA/ pCDNA3.1 | IV | 100 μg of plasmid DNA constructs. Boost 2 wk apart and 4wk after with recombinant bacilli, *Salmonella,* expressing LACK | Needle | Mouse (Balb/c) | - | Th1 response (IFN-γ and IgG2a) | Partial Protection | [78] |
|  | 20. *L. major* | Subunit:  LACK | DNA/ MIDGE | ID | 54.8 μg of plasmid DNA constructs. Boost 2 wk after. | Needle | Mouse (Balb/c) | - | Th1 response | Strong Protection | [79] |
|  | 21. *L. major* | Subunit:  CPB and CPA (cysteine proteinases type I and II) | Protein | SC | 40 μg rCPB or rCPA. Boost 3 wk after. | Needle | Mouse (Balb/c) | poloxamer 407 | Th1 response (IFN-γ) | rCPB - Partial Protection rCPA - No protection | [80] |
|  | 22. *L. major* | Subunit:  rCPB-CPA (hybrid protein) | Protein | SC | 30 μg rCPB-CPA. Boost 3 wk after. | Needle | Mouse (Balb/c) | poloxamer 407 | Th1 response (IFN-γ) | Partial protection | [81] |
|  | 23. *L. major* | Subunit:  CPB and CPA | DNA/pCB6 | IM | 100 μg of plasmid DNA constructs, single or together. Boost 1 month apart. | Needle | Mouse (Balb/c) | - | Th1 response (IFN-γ) | Partial protection | [82] |
|  | 24. *L. major* | Subunit:  PSA2 | Protein | IP | 3 μg of protein, 3 doses, 2wk interval. | Needle | Mouse (Balb/c) | *Corynebacterium*  *parvum* | Th1 response (IFN-γ) | PSA2 from E. coli - no protection; PSA2 from L. mexicana - strong protection | [83] |
|  | 25. *L. major* | Subunit:  PSA2 | Protein (in ISCOM) | IP | 2 μg of iscom, twice, 2 wk intervals | Needle | Mouse (C3H/He) | *Corynebacterium*  *parvum* | Th1 response (IFN-γ) | No protection | [84] |
|  | 26. *L. major* | Subunit:  Histone H1 | Protein (long and short peptides) | SC | 100 μg of protein, twice. | Needle | Mouse (Balb/c) | IFA + IL-12 exogenous | Not evaluated (protection was analyzed by lesion score) | Partial Protection | [85] |
|  | 27. *L. major* | Subunit:  Histone H2B | Protein (entire or truncated) | SC | 25 μg of protein twice, 2 wk intervals. | Needle | Mouse (Balb/c) | CpG | Not evaluated (protection was analyzed by lesion score and parasite burden) | aminoterminal - Partial protection; carboxiterminal - No protection | [86] |
|  | 28. *L. infantum* | Subunit:  Ribosomal P0 (LiP0) | Protein and DNA/pCDNA3 | SC | 100 μg of of plasmid DNA construct or 10 μg rLiP0, three times, 2 wk intervals. | Needle | Mouse (Balb/c and C57BL/6) | CpG ODN | Th1 response  (IFN-γ and ↑ IgG2 | Partial protection | [87] |
|  | 29. *L. infantum* | Subunit:  Ribosomal P0 (LiP0) | Protein and DNA/pCDNA3 | ID (protein) IM (DNA) | 100 μg of of plasmid DNA construct or 5 μg rLiP0, three times, 2 wk intervals | Needle | Mouse (Balb/c and C57BL/6) | - | Mixed Th1/Th2 response | Partial protection | [88] |
|  | 30. *L. infantum* | Subunit:  Histones H2A, H2B, H3 and H4 | DNA/pCDNA3 | SC | 80 μg of of plasmid DNA constructs alone or combination, three times | Needle | Mouse (Balb/c) | - | Th1 response  (IFN-γ) | Partial protection | [89] |
|  | 31. *L. infantum* | Subunit:  Histones H2A, H2B, H3 and H4 | DNA/pCDNA3 | IM | 50 μg of of each plasmid DNA constructs, together, three times, 2 wk intervals. | Needle | Mouse (Balb/c) | - | Th1 response  (IFN-γ) | Strong protection | [90] |
|  | 32. *L. infantum* | Subunit:  LiP2a and LiP2b | Protein  DNA/pCDNA3 | SC | 100 μg of plasmid DNA construct alone or 2 μg of rLiP2a or rLiP2b. Prime-boost with DNA first and rLiP2a or rLiP2b later. | Needle | Mouse (Balb/c) | CpG ODN (Protein) | mixed Th1/Th2 immune response | No protection | [91] |
|  | 33. *L. major* | Subunit:  LmSTI1 and TSA | Protein | SC | 10 μg of recombinanat proteins, alone or together. Boost 3 wk later. | Needle | Mouse (Balb/c) and Primate -Monkey | IL-12 exogenous | Th1 response | Strong protection | [92] |
|  | 34. *L. major* | Subunit:  LmSTI1 and TSA | DNA/pCDNA3 | IM | 100 μg of plasmid DNA construct, three times, 1 month apart. | Needle | Mouse (Balb/c) | - | Th1 response; CTL (onlyTSA) | Strong protection | [93] |
|  | 35. *L. major* | Subunit:  LeIF | Protein | SC | 50 μg of recombinant protein. Boost 10 μg 3 wk later. | Needle | Mouse (Balb/c) | - | Th1 response | Partial protection | [94] |
|  | 36. *L. major* | Subunit:  LeIF, LmSTI1 and TSA fusioned (Leish-111f polyprotein) | Protein | SC | 10 μg of the Leish-111f ( polyprotein containing LmSTI1, TSA and LeIF fusioned). Boost 3 wk later. | Needle | Mouse (Balb/c) | MPL-SE or Ribi  529-SE | Th1 response | Partial protection | [95] |
|  | 37. *L. major* | Subunit:  LeIF, LmSTI1 and TSA fusioned (Leish-111f polyprotein) | Protein | SC | 10 μg of the Leish-111 f ( polyprotein containing LmSTI1, TSA and LeIF fusioned), three times, 3 wk apart | Needle | Mouse (Balb/c) | MPL-SE | Th1 response  (IFN-γ) | Partial protection | [96] |
|  | 38. *L. major* | Subunit:  TRYP (TSA) | DNA/? | IM | 100 μg of plasmid DNA construct. Boost 4 wk later with 10^8^ pfu of MVA expressing TRYP | - | Dog | - | Th1 response | Not Evaluated | [97] |
|  | 39. *L. major* | Subunit:  LACKp24, TSA, LmSTI1 and CPa | DNA/ pCMV3ISS | IM | Cocktails with different plasmid DNA construct (50 μg each one). | Needle | Mouse (Balb/c) | - | Th1 response | Partial protection | [98] |
|  | 40. *L. major* | Subunit:  LRP (*Leishmania major* ribosomal proteins) | Protein (purified from promastigotes) | SC | 12 μg of protein. Boost 2 and 4 wk later. | Needle | Mouse (Balb/c) | CpG-ODN | Th1 response | Partial protection | [99] |
|  | 41. *L. major* | Killed (heat) | - | SC | 50 μg of protein extract. Boost 2 wk later. | Needle | Mouse (Balb/c) | Imiquimod and R848  (Toll-like receptor agonists) | Th1 response | Partial protection | [100] |
|  | 42. *L. infantum* | Subunit:  LACK | DNA/ | ID | 100 μg of plasmid DNA construct. Boost with 10^7^ recombinant virus expressing LACK 2wk later. | Needle | Mouse (Balb/c) | α-galactosylceramide  (aGalCer) | Th1 and CTL responses | Partial protection | [101] |
|  | 43. *L. major* | Dendritic cells pulsed with *L. major* lysate |  | SC | 5 x 10^4^ cells/animal | Needle | Mouse (Balb/c) | - | Th1 response | Partial protection | [102] |
|  | 44. *L. major* | Killed (ALM – autoclaved L major) |  | IM | 3.6 mg of ALM | Needle (ID leishmanin antigen, Pasteur Institute, Iran) | Human (fase II) | BCG or Alum | Not evaluated | Not Evaluated | [103] |
|  | 45. *L. major* | Live |  | ID | 10^4^ Lm metacyclic promastigotes | Needle | Mouse (C57BL/6) | Cpg 1826 | Th1 response | Strong Protection | [104] |
|  | 46. *L. major* | Subunit:  SPase(signal peptidase type I) | Protein and DNA/pCDNA3.1 | SC | DNA/DNA: 100 μg of plasmid DNA, boost 3 and 6 wk later. Protein/Protein: 30 μg of recombinat protein, boost 3 and 6 wk later.  DNA/Protein: 100 μg of plasmid DNA, boost 3 and 6 wk later with 30 μg of recombinant protein | Needle | Mouse (Balb/c) | Cpg ODN and Montanide 720 (immunization protein) | Th1 response | Partial protection | [105] |
|  | 47. *L. major* | Live | Whole parasite | ID (ear) or SC (footpad) | 10^4^ L. Major V1 metacyclic promastigotes | - | Mouse (C57BL/6) | - | Th1 response | Strong protection | [106] |
|  | 48. *L. major* | Killed (autoclaved) | *Leishmania* proteins | ID | 200 μg of proteins. Single dose | - | Dog (phase III  182 dogs) | Alum + BCG | Not evaluated, but probably Th1 response. Efficacy of vaccine was 70% | Partial protection | [107] |
|  | 49. *L. major* | Killed (autoclaved) | *Leishmania* proteins | ID | 10 μg, 100 μg, 200 μg or 400 μg of proteins. | - | Human (phase II? 24 volunteers) | Alum + BCG | DTH response | Not Evaluated | [108] |
|  | 50. *L. major* | Live, attenuated (*lpg2-* This gene encodes the enzyme involved  in the transport of GDP mannose to the Golgi for the assembly  of disaccharide-phosphate repeats of LPG and other  phosphoglycan-containing molecules | Whole parasite | ID | 5 x 10^6^ attenuated parasites | Needle | Mouse (Balb/c) | - | Th1 response | Strong Protection | [109] |
|  | 51. *L. major* | Live, attenuated |  | SC | 5 x 10^6^ of *L. mexicana* | Needle | Mouse (Balb/c) | - | Not evaluated (protection was analyzed by lesion size) | Partial protection | [110] |
|  | 52. *L. major* | Live, attenuated *dhfr-ts-* (-/-) mutant organism |  |  | 10^8^ attenuated *L. major* | Needle | Primate - Monkey | - | Th1 immune response | Partial protection | [111] |
|  | 53. *L. major* | Subunit:  Meta 1 | Protein and DNA/pVX | SC (Protein) IM (DNA) | 30 μg of protein, 3 doses with 2 wk interval or 50 μg of DNA construct, , 3 doses with 2 wk interval | Needle | Mouse (Balb/c) | CFA and IFA (for protein) | Th2 immune response | No protection | [112] |
|  | 54. *L. major* | Killed (ALM) | *Leishmania* proteins | SC | 50 μg of protein (ALM). Boost 2 wk later. | Needle | Mouse (Balb/c) | CPG ODN | CTL and Th1 immune response | Partial Protection | [113] |
|  | 55. *L. major* | Killed (ALM) | *Leishmania* proteins | ? | 1 mg of proteins | Needle | Primate - Monkey | BCG + Alum | Th1 response | Partial protection | [114] |
|  | 56. *L. major* | Killed (ALM) | *Leishmania* proteins | ID | 1 mg of proteins; single dose. | - | Primate – Human (Phase III) | BCG | Th1 response | Partial protection | [115] |
|  | 57. *L. major* | Killed (ALM) | *Leishmania* proteins | ID | 1 mg of proteins; single dose. |  | Primate - Monkey | Recombinant IL-12 | Th1 response | No protection | [116] |
|  | 58. *L. major* | Killed (ALM) | *Leishmania* proteins | ID | 2 doses with 4 wk interval | - | Primate – Human (Phase III – Clinical trial) | BCG | Not evaluated. | Partial protection | [117] |
|  | 59. *L. major* | Subunit:  ACP (amastigote cysteine proteinase) | Protein (isolated and purified) | IP | 5 μg of protein. Boost 1 month later. | Needle | Mouse (Balb/c) | CFA and IFA | Th1 response | Partial protection | [118] |
|  | 60. *L. major* | Subunit:  GP63 | Protein | SC | 5 μg of protein/dose. Many schedules of immunization. | Needle | Mouse (CBA) | CFA, MDP or BCG | Cellular response ? | Partial protection | [119] |
|  | 61. *L. major* | Killed (ALM) | *Leishmania* proteins | ID | 1 mg of proteins; single dose | - | Primate – Human (Phase III – Clinical trial) | BCG | Not evaluted. | Partial protection | [120] |
|  | 62. *L. major* | Epidermal Langerhans cells pulsed with *L. major* (Immunotherapy) | - | IV | 4 x 10^5^ cells. | Needle | Mouse (Balb/c) | - | Th1 response | Strong protection | [121] |
|  | 63. *L. major* | Subunit:  Recombinant GP63 | Protein | ID | 50 μg of protein, 3 dose with 2 wk interval. | - | Primate - Monkey | BCG | DTH and Th1 response | Partial protection | [122] |
|  | 64. *L. major* | Killed, irradited |  | IV |  |  | Mouse (Balb/c) | - | Partial protection | Partial protection | [123] |
|  | 65. *L. major* | Killed, irradited |  | IV or SC | 2 x 10^7^ of promastigotes, once, twice or four times. | Needle | Mouse (Balb/c) | Dehydration-rehydration vesicle (DRV) liposomes | Not evaluated. Protection was analyzed by lesion size. | SC - Partial protection; IV - No protection | [124] |
|  | 66. *L. major* | Subunit:  SLA |  | IP | 100 μg of protein Boost 1 wk later with protein alone. | Needle | Mouse (Balb/c) | *Corynebacterium parvum* | DTH and Humoral response | No protection | [125, 126] |
|  | 67. *L. major* | Killed |  | IP, IV or SC | 1 x 10^6^ promastigotes |  | Mouse (Balb/c) | - | Not evaluated. Protection was analyzed by lesion size | Partial protection | [127] |
|  | 68. *L. major* | Killed or nonpathogenic living |  | IP, IV or SC | 24 x 10^6^ promastigotes | Needle | Mouse (Balb/c) | - | Partial protection | IP and IV: Partial protection; SC: No protection | [128] |
|  | 69. *L. major* | Live, attenuated (null mutant, *lpg1-* [lipophosphoglycan]) |  | SC | 10^6^ parasites | - | Mouse (Balb/c) | - | Not evaluated. Protection was analyzed by lesion size) | Not Evaluated | [129] |
|  | 70. *L. major* | Live, attenuated (null mutant, *lpg2-* [lipophosphoglycan]) |  | SC | 10^6^ parasites | - | Mouse (Balb/c) | - | Not evaluated. Protection was analyzed by lesion size) | Not Evaluated | [130, 131] |
|  | 71. *L. major* | Live, attenuated (null mutant *dhfr-ts-* [dihydrofolate reductasethymidylate  Synthas])] |  | SC, IV or IM | 10^2^ – 10^6^ parasites | Needle | Mouse (Balb/c) | - | Not evaluated. Protection was analyzed by lesion size) | Partial protection | [132] |
|  | 72. *L. major* | Killed (irradiated) |  | IV | 2 x 10^6^ parasites, four weekly doses. | - | Mouse (Balb/c) |  | Humoral response | Partial protection | [128] |
|  | 73. *L. major* | Killed (irradiated) |  | IV | 2 x 10^7^ promastigotes, four weekly doses | Needle | Mouse (Balb/c) | - | Humoral response | Partial protection | [133] |
|  | 74. *L. major* | Live, attenuated (phosphomannomutase  Deficient) |  | SC | 5 x 10^6^ parasites (Δ PMM) | Needle | Mouse (Balb/c | - | Cellular response | Partial protection | [134] |
|  | 75. *L major* | Killed (ALM) | *Leishmania* proteins | SC | 50 mg, three times, at 2 week intervals. | Needle and Vector (Experimental) | Mouse (C57BL/6) | CpG ODN | Netrophilic response elicited by vector bite decreases parasite-specific immune responses | Partial protecton with needle challenge and no protection with vector challenge | [135] |
|  | 76. *L major* | Subunit: PpSP 12,14,15, 28, 30, 32, 36, 42, 44 and PsAg5 (proteins from the vector salivary gland) | DNA/ VR2001-TOPO | ID | 5 μg, three times, at 2 week intervals. | Needle (salivary gland homogenate and L. major metacyclics) | Mouse (C57BL/6) | - | Th1 response (IFN-γ) elicited only by PpS15 | Mixed response, depending on the polypeptides | [136] |
|  | 77. *L major* | Live, Recombinant (*tk–cd*+*/*+) |  |  | 2 x 10^6^ promastigotes (wt or *–cd*+*/*+). Treatment for 14 consecutive  Days receiving i.p. injections  of GCV daily. |  | Mouse (Balb/c) | - | Not evaluated. Protection was evaluated by healed lesions |  | [137] |
| L. tropica | 1. Killed |  | Proteins | ID | 2 x 10^6^ promastigotes (nao consegui paper completo) | - | Primate – Human (Clinical trial) | - | Not evaluated. | Not evaluated. | [138] |
| L. guyanensis |  |  |  |  |  |  |  |  |  |  |  |
| L. braziliensis | 1. *L braziliensis* | Subunit:  LACK, LmSTI1, LeIF, and TSA | Protein and DNA/pCDNA3 | SC (protein)  IM (DNA) | 25 μg of each recombinant protein administers alone or together. 2 boosts with 2 wk apart.  50 μg of plasmid DNA construct. Boosts 2wk and 4 wk after the 1^st^ immunization. | Needle | Mouse (Balb/c) | CpG ODN 1826 + aluminum hydroxide | No protection | No protection | [139] |
|  | 2. *L braziliensis* and *L. amazonensis* | Killed | Protein (crude extract) | SC | 200 μg of extract protein. 3 doses at 4 wk interval. | - | Dog | BCG | Humoral immune response | Not Evaluated | [140] |
|  | 3. *L braziliensis*, *L. amazonensis* and *L. mexicana* | Killed | Protein (crude extract) | IM | 1.2 mg of proteins and boost 7 days later with 800 μg of protein. | - | Primate – Human (Clinical trial) | *Corynebacterium parvum* | Humoral immune response | Not Evaluated | [141] |
|  | 4. *L braziliensis* | Killed | Protein (crude extract) | ID | 2 x 10^6^ promastigotes, 3 doses weekly. | Needle | Rat | - | Not evaluated (protection was analyzed by lesion) | Partial Protection | [142] |
|  | 5. *L braziliensis* | Live, Attenuated (centrin gene-deleted null mutant) |  | IV | 3 x 10^6^ parasites | Needle | Mouse (Balb/c) | - |  | Partial protection | [52] |
| L. mexicana | 1. *L. major* | Subunit:  Native Gp63 | Protein | SC | 5 μg of native Gp63/dose. 2^nd^ immunization 4 wk after the 1^st^. | Needle | Mouse (Balb/c or CBA/Ca) | Lipossome | Not evaluated (protection was analyzed by lesion score) | Partial Protection | [143] |
|  | 2. *L. major* | Recombinant bacilli:  BCG expressing Gp63 cloned in pMV262 | Recombinant bacilli | SC | 10^6^ recombinant bacilli | Needle | Mouse (Balb/c) | - | Not evaluated (protection was analyzed by lesion score) | Partial Protection | [67] |
|  | 3. *L. mexicana* | Recombinant bacilli:  *Salmonella* expressing Gp63 | Recombinant bacilli | Mucosal (oral) | 1 x 10^9^ recombinant bacilli. Boost after 3 wk. | Needle | Mouse (Balb/c) | - | CTL response; Th1 response (IFN-γ) | Strong Protection | [144] |
|  | 4. *L. mexicana* | Subunit:  Gp63, LACK and CPb cloned separately | DNA/VR1012 | IM | 100 μg of plasmid DNA constructs single or together. Boost 2 wk after. | Needle | Mouse (Balb/c) | - | Th1 response | Partial Protection | [145] |
|  | 5. *L. mexicana* | Subunit:  Gp63 and CPb cloned separately | DNA/VR1012 | IM | 100 μg of plasmid DNA construct. | Needle | Mouse (Balb/c) | - | ? | Partial Protection | [146] |
|  | 6. *L. major* | Subunit:  LACK | DNA/VR1012 | IM | 100 μg of plasmid DNA constructs | Needle | Mouse (Balb/c) | - | ? | No protection | [146] |
|  | 7. *L. amazonensis* | Subunit:  Gp46 | DNA/VR1012 | IM | 100 μg of plasmid DNA constructs | Needle | Mouse (Balb/c) | - | ? | Partial Protection | [146] |
|  | 8. *L. mexicana* | Live, attenuated |  | SC | 5 x 10^6^ of *L. mexicana* | Needle | Mouse (Balb/c) | - | Not evaluated (protection was analyzed by lesion size) | Partial Protection | [110] |
|  | 9. L mexicana | Subunit:  rCP5 (cysteine protease) r GP63 and rMBAP 9 (membrane-bound acid phosphatase) | Protein | SC or IP | 2.5 μg of mixture of the recombinant proteins,  2 doses with 1 wk interval. | Needle | Mouse (C57BL/6) | rIL-12, Detox, 4*-monophosphoryl lipid A, QS-21, BCG and *Corynebacterium parvum* | Th1 response | rMBAP 9 - No protection, rCP5 and rPG3 - Partial protection, All - Partial protection, best adjuvants: rIL-12 and Detox | [147] |
|  | 10. L mexicana | Killed | *Leishmania* proteins | ID | 3 doses with 6-10 wk interval | - | Primate – Human (Clinical Trial - phase | BCG | Humoral response | Partial Protection | [148] |
|  | 11. L mexicana | Killed | *Leishmania* proteins | ID | 3 doses with 6-10 wk interval | - | Primate – Human (Clinical Trial - phase | BCG | Th1 response | Partial Protection | [149] |
|  | 12. *L braziliensis*, *L. amazonensis* and *L. mexicana* | Killed | Protein (crude extract) | IM | 1.2 mg of proteins and boost 7 days later with 800 μg of protein. | - | Primate – Human (Clinical trial) | *Corynebacterium parvum* | Humoral response | Not Evaluated | [141] |
|  | 13. L mexicana | Subunit:  Microsomal preparation (Pol-F) | Protein | SC | 10 μg of protein | Needle | Mouse | BCG | Humoral response | Partial Protection | [150] |
|  | 14. L mexicana | Live, attenuated (null mutant, *lpg2-* [lipophosphoglycan]) |  | SC | 10^5^ or 10^6^ parasites | - | Mouse (Balb/c) | - | Not evaluated. Protection was analyzed by lesion size | No protection (not safe) | [151] |
|  | 15. L mexicana | Live, attenuated (*cpa-* and *cpb-* mutant) |  | SC | 5 x 10^6^ parasites | Needle | Mouse (Balb/c and CBA/Ca) | - | Th1 response | Partial protection | [152] |
|  | 16. *L Mexicana, L.major and L. donovani* | Subunit: synthetic glycovaccine  (glycans found in promastigote secretory gel) | Synthetic glycans conjugated with Recombinant tetanus toxin fragment C | SC | 5 μg of synthetic glycovaccine twice, at 2-week intervals | Vector; experimental | Mouse (Balb/c) | CpG (ODN) or not | Not evaluated. Protection was analyzed by reduction in lesion size and parasite burden | Partial protection to sand fly bite. No protection to needle challenge | [153] |
| *L. amazonensis* | 1. *L. infantum* | Subunit:  LACK/LaAg | Protein and DNA/ pCI-neo | Mucosal (nasal) | 10 μg of of plasmid DNA construct or 10 μg of protein. Boost 2 wk after. | Needle | Mouse (Balb/c) | - | Th1 response (IFN-γ) | partial protection for soluble intranasal LaAg and LACK DNA | [154] |
|  | 2. *L. amazonensis* | Recombinant virus:  GP46/M2 | Recombinant virus:  Vaccinia virus expressing | IP | 10^7^ pfu of recombinant vaccinia virus. Boost 4 wk later. | Needle | Mouse (Balb/c) | - | Mixed Th1 e Th2 responses | Partial Protection | [155] |
|  | 3. *L braziliensis* and *L. amazonensis* | Killed | Protein (crude extract) | SC | 200 μg of extract protein. 3 doses at 4 wk interval. | - | Dog | BCG | Humoral immune response | Not Evaluated | [140] |
|  | 4. *L. donovani* | Subunit:  A2 | DNA | IM | 100 μg of plasmid DNA constructs. Boost 3 and 6 wk later. | Needle | Mouse (Balb/c) | - | Th1 response | Partial Protection | [59] |
|  | 5. *L. amazonensis* | Subunit:  (LPG) lipophosphoglycan | Protein (isolated from L. amazonensis lysate) | Mucosal (intranasal) | 10 μg of protein. Boost 1 wk later. | Needle | Mouse (Balb/c) | - | Th1 response | Partial Protection | [156] |
|  | 6. *L. amazonensis* | Subunit:  SLA and MEA (membrane-enriched) | Protein | ID | 250 μg of protein (SLA-MEA). | Needle | Mouse (Balb/c) | ONO-4007 | Th1 response | Partial Protection | [157] |
|  | 7. *L. amazonensis* | Subunit:  SLA | Protein | SC | 10 μg of protein. Boost 15 and 30 days later | Needle | Mouse (Balb/c) | KM+ lectin | Th1 response | Partial Protection | [158] |
|  | 8. *L. amazonensis* | Killed (Leishvacin®) | *Leishmania* proteins | IM | 360 μg of merthiolate-killed L amazonensis, 3 doses with 3 wk interval | - | Human (phase III) | - | Not evaluated. Formulation afe but not efficacious | No protection | [159] |
|  | 9. *L. amazonensis* | Killed (Leishvacin®) | *Leishmania* proteins | ID | 100 μL of Leishvacin®, 2 doses with 8 wk interval | - | Human (phase III) | BCG | No significant protection | No protection | [160] |
|  | 10. *L. amazonensis* | Lag (antigens from whole promastigotes) | *L. amazonensis*  proteins | Mucosal (oral) | 100 μg of protein, 3 doses with 2 wk interval. | Needle | Mouse (Balb/c and C57BL/6) | - | Th1 response | Partial Protection | [161] |
|  | 11. *L. amazonensis* | Killed | *Leishmania* proteins | IM or ID | 3 doses with 3 wk interval. | - | Human (phase II) | - | Th1 response | Not Evaluated | [162] |
|  | 12. *L. amazonensis* | Killed (autoclaved) | *Leishmania* proteins | IM | 1.5 mL (?) 2 doses with 3 wk interval. | - | Human (Clinical trial) | ? | Th1 response | Not Evaluated | [163] |
|  | 13. *L braziliensis*, *L. amazonensis* and *L. mexicana* | Killed | Protein (crude extract) | IM | 1.2 mg of proteins and boost 7 days later with 800 μg of protein. | - | Primate – Human (Clinical trial) | *Corynebacterium parvum* | Humoral immune response | Not Evaluated | [141] |
|  | 14. L major | Live, attenuated (null mutant *dhfr-ts-*) |  | IV or SC | 10^4^, 10^6^ or 10^8^ promastigotes | Needle | Mouse (Balb/c and C57BL/6) | - | Not evaluated. Protection was analyzed by lesion size. | Partial Protection | [164] |

**^1^**Antigen from one species of Leishmania is used to confer protection against another

* Not Available
